# Novel *P-n* Li_2_SnO_3_/g-C_3_N_4_ Heterojunction With Enhanced Visible Light Photocatalytic Efficiency Toward Rhodamine B Degradation

**DOI:** 10.3389/fchem.2020.00075

**Published:** 2020-02-11

**Authors:** Yuanyuan Li, Meijun Wu, Yaoqiong Wang, Qimei Yang, Xiaoyan Li, Bin Zhang, Dingfeng Yang

**Affiliations:** ^1^Department of Biological and Chemical Engineering, Cooperative Innovation Center of Lipid Resources and Children's Daily Chemicals, Chongqing University of Education, Chongqing, China; ^2^College of Chemistry and Chemical Engineering, Chongqing University of Technology, Chongqing, China; ^3^National ad Local Joint Laboratory of Traffic Civil Engineering Materials, Department of Materials and Engineering, Chongqing Jiaotong University, Chongqing, China; ^4^Analytical and Testing Center of Chongqing University, Chongqing, China

**Keywords:** Li_2_SnO_3_, g-C_3_N_4_, *p-n* heterojunction, photocatalysis, Rhodamine B, Photoelectrochemistry

## Abstract

The design of highly efficient and stable photocatalysts to utilize solar energy is a significant challenge in photocatalysis. In this work, a series of novel *p-n* heterojunction photocatalysts, Li_2_SnO_3_/g-C_3_N_4_, was successfully prepared via a facile calcining method, and exhibited superior photocatalytic activity toward the photodegradation of Rhodamine B solution under visible light irradiation as compared with pure Li_2_SnO_3_ and g-C_3_N_4_. The maximum kinetic rate constant of photocatalytic degradation of Rhodamine B within 60 min was 0.0302 min^−1^, and the composites still retained excellent performance after four successive recycles. Chemical reactive species trapping experiments and electron paramagnetic resonance demonstrated that hydroxyl radicals (·OH) and superoxide ions ($\cdot {\text{O}}_{2}^{-}$) were the dominant active species in the photocatalytic oxidation of Rhodamine B solution, while holes (h^+^) only played a minor role. We demonstrated that the enhancement of the photocatalytic activity could be assigned to the formation of a *p-n* junction photocatalytic system, which benefitted the efficient separation of photogenerated carriers. This study provides a visible light-responsive heterojunction photocatalyst with potential applications in environmental remediation.

## Introduction

The presence of harmful and toxic substances in aqueous solution poses severe risks to human health and ecosystems. The purification of waste water is an urgent priority and a major research theme in environmental science (Shannon et al., 2008; Damasiewicz et al., 2012). As a promising technique for oxidation of pollutants, semiconductor-based photocatalysis, which uses solar energy to drive chemical reactions, has an important role in environmental remediation (Chen et al., 2010). Among semiconductor photocatalysts, layered metal oxides have attracted much attention owing to their low cost, photostability, and oxidation capability (Osada and Sasaki, 2009; Lei et al., 2014; Haque et al., 2018).

Recently, the semiconductor Li_2_SnO_3_ has been applied as a UV light-responsive photocatalyst with excellent photocatalytic performance and chemical stability (Li Y. Y. et al., 2019). As a state-of-the-art layered photocatalyst, the compound features a conventional [Li_1/3_Sn_2/3_O_2_]^−^ anion layered structure in the *a*-*b* planes, while the rest of the Li^+^ cations embed in the interlayer spaces to balance the charge (Howard and Holzwarth, 2016). The resulting charge density distribution in space generates an electrostatic field perpendicular to the laminar direction, promoting the separation of photo-induced carriers to drive photocatalysis. In addition, the valence band edge of Li_2_SnO_3_ is positive enough to oxidize organic pollutants. However, similar to many other oxide semiconductors, Li_2_SnO_3_ can only absorb UV light, while its harvesting of solar energy is poor owing to its wide intrinsic optical band gap (~3.7 eV), limiting its photocatalytic activity. Constructing visible light-responsive Li_2_SnO_3_-based heterojunction photocatalysts to make full use of sunlight is thus an important goal. This kind of system, where a heterojunction is formed between a visible light- and a UV light-responsive photocatalyst, has received some attention in recent years (Pan et al., 2012; Li et al., 2017; Wu et al., 2017; Liu et al., 2018; Qiao et al., 2018; Wang et al., 2018a; Hafeez et al., 2019). For instance, ZnFe_2_O_4_/TiO_2_ heterojunctions exhibited outstanding photocatalytic degradation of bisphenol A under visible light irradiation (Nguyen et al., 2019). A CdS/SrTiO_3_ nanodots-on-nanocubes heterojunction presented excellent visible light photocatalytic performance for oxidation of H_2_ (Yin et al., 2019). Notably, Dong et al. successfully synthesized an insulator-based core-shell SrCO_3_/BiOI heterojunction structure, and this nanocomposite displayed an unprecedentedly high photocatalytic NO removal performance (Wang et al., 2018b). Therefore, the heterojunction strategy clearly provides opportunities to utilize wide-band-gap semiconductors with excellent intrinsic photophysical properties as visible light-responsive photocatalysts.

Among the best known classes of such catalysts are *p-n* heterojunctions, which have been extensively studied to optimize their photocatalytic activity. Their catalytic mechanism is based on an internal electric field established at the interface of the *p-n* junction, which promotes the efficient separation of photogenerated carriers (Wen X. J. et al., 2017; Dong et al., 2018; Dursun et al., 2018; Wang et al., 2018; Zeng et al., 2019). The Mott-Schottky plots measured by electrochemistry demonstrate that Li_2_SnO_3_ is a *p*-type semiconductor. Therefore, to improve its photocatalytic performance, it is necessary to couple Li_2_SnO_3_ with *n-*type and visible light-responsive semiconductors to build *p-n* heterojunction systems, which would be able to simultaneously realize high utilization rates of solar energy and efficient separation of photogenerated carriers. Among numerous *n-*type photocatalytic semiconductors, g-C_3_N_4_ is a promising candidate for its tunable photo-response, and effective charge carrier transportation properties. As a photocatalyst, g-C_3_N_4_ has been widely investigated owing to its excellent properties including layered graphite-like structure, visible light-responsive band gap (~2.7 eV), facile preparation, low toxicity, and high photostability (Wang et al., 2009, 2019; Ong et al., 2016; Wen J. Q. et al., 2017; Lu et al., 2018; Zhang et al., 2018; Li X. B. et al., 2019). Furthermore, as an *n-*type semiconductor, g-C_3_N_4_ has been selected to be coupled with *p-*type semiconductors to enhance photocatalytic activity, such as in CuBi_2_O_4_/g-C_3_N_4_ (Guo et al., 2017), Bi_4_Ti_3_O_12_/g-C_3_N_4_ (Guo et al., 2016), and LaFeO_3_/g-C_3_N_4_ (Liang et al., 2017).

## Experimental Section

### Synthesis of G-C_3_N_4_, Li_2_SnO_3_, and Li_2_SnO_3_/g-C_3_N_4_ Heterojunction

g-C_3_N_4_ was prepared by annealing melamine in a muffle furnace. Briefly, 5 g melamine was heated in an closed crucible at a rate of 4.5°C/min to 560°C and maintained for 2 h. Then, the furnace was turned off and cooled to room temperature naturally. Pure Li_2_SnO_3_ was synthesized from a mixture of Li_2_CO_3_ and SnO_2_ with a molar ratio of 3.3/3.0. The mixed reactants were ground together within a mortar for 30 min. Then, the mixture was heated at 850°C for 6 h. The heterojunctions Li_2_SnO_3_/g-C_3_N_4_ (LSO-CN) with different mass ratios were prepared by a traditional solid state method. Samples with initial mass ratios of g-C_3_N_4_ to LSO-CN having values of 70, 80, 85, 90, and 95 wt% were prepared, and labeled as LSO-CN-70, LSO-CN-80, LSO-CN-85, LSO-CN-90, and LSO-CN-95, respectively. Taking LSO-CN-85 as an example, 0.03 g of Li_2_SnO_3_ powder, 0.17 g of g-C_3_N_4_ and 1 mL ethanol were mixed, and ground together for 10 min. The resultant mixture was heated at 500°C for 2 h in a covered crucible.

### Characterization

Powder X-ray diffraction (PXRD) was performed on a PANalytical X'pert powder diffractometer equipped with a PIXcel detector and with CuKα radiation (40 kV and 40 mA). The scanning step width of 0.01° and the scanning rate of 0.1° s^−1^ were applied to record the patterns in the 2θ range of 6–90°. A JEOL JSM-6700F field emission scanning electron microscope (SEM) was employed to investigate the surface morphologies. The transmission electron microscopy (TEM) and high-angle annular dark field (HAADF) images and energy-dispersive spectra (EDS) of Li_2_SnO_3_ were recorded by a Talos F200S G2 Microscope to characterize the microstructures of the samples. The UV-vis diffuse reflectance spectroscopy (UV-vis DRS) data were collected at room temperature using a powder sample with BaSO_4_ as a standard on a Shimadzu UV-3150 spectrophotometer over the spectral range 200–800 nm. The Fourier transform infrared (FT-IR) spectra were obtained by using a Nicolet 360 spectrometer with a 2 cm^−1^ resolution in the range of 500–4,000 cm^−1^. Fluorescence spectra were measured on a Hitachi fluorescence spectrophotometer F-7000 to detect the concentration of, in which the fluorescence emission spectrum (excited at 316 nm) of the solution was measured every 15 min during the photocatalytic reaction. The solid-state photoluminescence (PL) spectra were acquired using a Fluorolog-TCSPC luminescence spectrometer with an excitation wavelength of 325 nm. In the electron paramagnetic resonance(EPR) experiments, 10 mg of LSO-CN-85 sample and 40 μL of 5,5 Dimethyl-1-pyrroline N-oxide (DMPO) was dispersed into 1 mL of deionized water (DMPO-·OH) or methol (DMPO-·${\text{O}}_{2}^{-}$), and then irradiated with visible light (λ > 420 nm) for 5 and 10 min, respectively. Electrochemical measurement was conducted on a CHI 660E workstation. A Pt plate, a calomel electrode and sample LSO-CN-85 coated on indium tin oxide (ITO) served as the counter electrode, reference electrode and working electrode, respectively, in a three-electrode cell. Electrochemical impedance spectroscopy (EIS) was carried out using an alternating voltage of 5 mV amplitude over the frequency range of 10^5^-0.1 Hz with an open circuit voltage in 0.5 M Na_2_SO_4_. For the analysis of transient photocurrent responses, a 300-W Xe lamp (cut-off λ > 420 nm; CEL-HXF300, Beijing Aulight) and Na_2_SO_4_ were employed as the light source and electrolyte, respectively. The Mott-Schottky curves were measured in Na_2_SO_4_ solution and the amplitude perturbation was 5 mV with frequencies of 1,000 Hz.

### Photocatalytic Activity Measurement

The photocatalytic performance of the LSO-CN composites was evaluated by the degradation of RhB. The light irradiation source was the above-mentioned Xe lamp with a filter (λ ≥ 420 nm) laid on the top of the reaction vessel. The light source was kept 7 cm away from the top of the reaction vessel and the reactant solution was maintained at room temperature by providing a flow of cooling water during the photocatalytic reaction. Before irradiation, the photocatalyst powder (30 mg) and RhB solution (10 mg L^−1^, 100 mL) were fully stirred in the dark for 1 h to establish the adsorption–desorption equilibrium. Then, the reaction was exposed to the light, and 5 mL samples of the suspension were extracted at a given time interval and separated by centrifugation. The concentration of RhB solution was determined by UV-vis spectrometry at its maximum absorption peak of 554 nm. Typically, the trapping experiments of active species were carried out as follows: 30 mg LSO-CN-85 and dye solution (10 mg/L, 100 mL) were mixed. Then, 10 mL 2-propanol (IPA), 0.1 mmol disodium ethylenediaminetetraacetic acid (EDTA), and 0.1 mmol ascorbic acid were added in sequence to trap radicals, holes (h^+^) and radicals, respectively. Additionally, trapping experiments under fluorescence spectroscopy were carried out as follows: 30 mg LSO-CN-85 and 8.3 mg terephthalic acid (TA) were dissolved in 100 mL NaOH solution (2 mmol/L), then the solution was stirred for 60 min in the dark and irradiated by the 300-W Xe lamp.

### Results and Discussion

**Figure 1:**
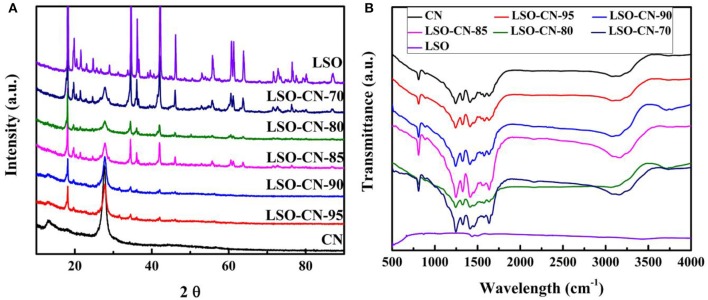
**(A)** XRD patterns, **(B)** FT-IR spectra of g-C_3_N_4_, Li_2_SnO_3_, and LSO-CN heterojunctions.

The crystallographic structure and phase purity of the as-synthesized samples were confirmed by PXRD. As presented in Figure 1, one small peak at 13.1 and one strong peak at 27.4 for pure g-C_3_N_4_ were assigned to the (100) and (002) crystal plane, respectively, in good accordance with previous reports (Hou et al., 2013). For Li_2_SnO_3_, the XRD pattern matched well with the monoclinic phase (JCPDS No. 00-031-0761). The two characteristic peaks of g-C_3_N_4_ gradually decreased in intensity with the increase of the Li_2_SnO_3_ content in the LSO-CN composites, whereas the peak intensity of Li_2_SnO_3_ strengthened gradually, reflecting the co-existence of Li_2_SnO_3_ and g-C_3_N_4_ in these heterojunctions. Further, the compositions of Li_2_SnO_3_, g-C_3_N_4_ and the LSO-CN heterojunction photocatalysts were confirmed by FT-IR. As shown in Figure 1B, for pure Li_2_SnO_3_, characteristic absorption peaks appeared at 519, 1,430, 1,495, and 3,435 cm^−1^, and the peak located at 519 cm^−1^ was assigned to the stretching vibration of Sn-O-Sn and Sn-O groups (Wang et al., 2012). In the FT-IR spectrum of g-C_3_N_4_, the peak located at 807 cm^−1^ was assigned to the breathing vibration mode of triazine units. The absorption peaks in the range of 1,000–1,800 cm^−1^ were ascribed to the C=N and aromatic C-N stretching vibration modes, whereas the peaks ranging from 3,000 to 3,500 cm^−1^ originated from the N-H stretching vibrations. The main characteristic peaks of the heterojunctions LSO-CN were similar to those of pure g-C_3_N_4_ because of the relatively weak vibration intensity of Li_2_SnO_3_. Notably, however, compared with the g-C_3_N_4_, the characteristic peaks at 1,241, 1,320, 1,413, and 1,631 cm^−1^ of sample LSO-CN-85 were shifted to higher wavenumbers, which indicated possible interfacial interactions involving electron transfer in these LSO-CN heterostructures (Figure S1).

**Figure 2:**
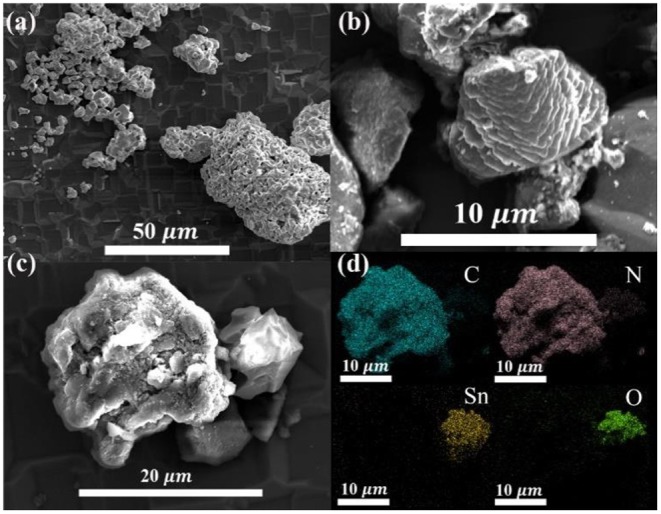
SEM micrographs of **(a)** Li_2_SnO_3_, **(b)** g-C_3_N_4_, **(c)** LSO-CN-85, and **(d)** element mapping of heterojunction LSO-CN-85.

SEM measurements were carried out to examine the morphology of the as-synthesized photocatalysts. Evidently, the as-prepared Li_2_SnO_3_ photocatalysts (Figure 2a) exhibited irregular bulk morphologies with an average particle length of ~6 μ m. Figure 2b presents the existence of large aggregates of g-C_3_N_4_ with a folded thin-sheet morphology. After combining Li_2_SnO_3_ and g-C_3_N_4_ into a heterojunction, irregular aggregates of Li_2_SnO_3_ were observed to adhere to g-C_3_N_4_ (Figure 2c), and the SEM-EDS element mapping showed a homogeneous distribution of Sn, O, C and N throughout the heterojunction (Figure 2d).

**Figure 3:**
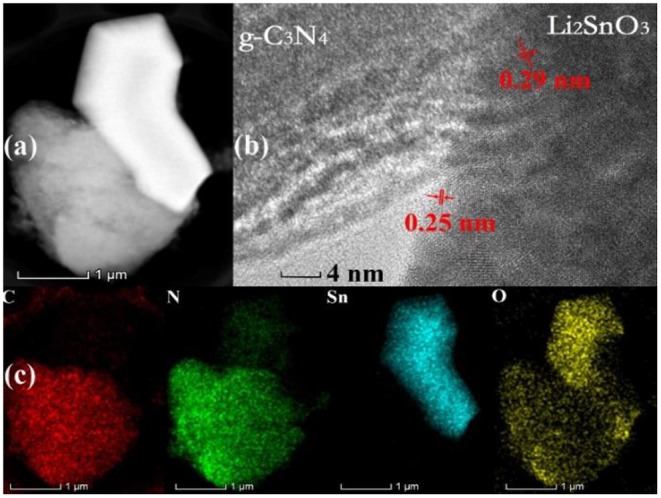
Heterojunction LSO-CN-85: **(a)** HAADF-TEM, **(b)** HRTEM, and **(c)** elemental mapping.

The intimate contact at the heterojunction between Li_2_SnO_3_ and g-C_3_N_4_ can be further observed in the representative HAADF-TEM image in Figure 3a. Meanwhile, the interface formed after the addition of Li_2_SnO_3_ into the LSO-CN-85 heterojunction can be clearly seen in the HRTEM image (Figure 3b). Notably, no distinct lattice fringes could be observed in g-C_3_N_4_ because of its low crystallinity, whereas distinct lattice fringes with a lattice spacing of 0.25 and 0.29 nm were found in Li_2_SnO_3_, which were ascribed to the (131) and (−113) planes, respectively. This kind of heterojunction system would be expected to reduce the recombination probability of photo-induced carriers and improve the photocatalytic activity. Additionally, TEM-EDS elemental mapping was performed to further authenticate the hybridization of the *p-*type and *n*-type semiconductors. As presented in Figure 3c, the elements Sn, O, C, and N were distributed uniformly across the assemblies, in good accordance with the results of SEM-EDS. In summary, the above analysis by powder XRD, FT-IR, SEM, and TEM manifested that a heterojunction interface was successfully formed in the composite between Li_2_SnO_3_ and g-C_3_N_4_.

**Figure 4:**
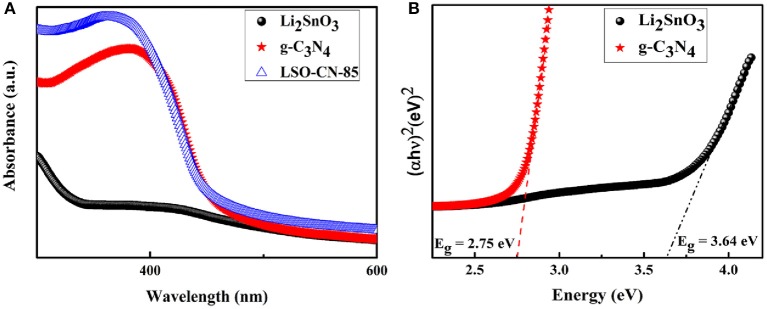
**(A)** UV-vis spectra of Li_2_SnO_3_, g-C_3_N_4_ and LSO-CN-85 **(B)** Plots of (α*hν*)^2^ vs. photon energy (*hν*) to calculate the band gap energies for Li_2_SnO_3_ and g-C_3_N_4_.

The light absorption ability of the as-prepared samples was determined via UV-vis reflectance spectroscopy to evaluate the optical band gaps. As shown in Figure 4A, pure Li_2_SnO_3_ presented a typical absorption edge at ~340 nm, and the estimated band gap energy *E*_g_ was about 3.64 eV (Figure 4B, black trace). For the pure g-C_3_N_4_ (Figure 4A, red trace), the absorption edge was extended to 451 nm, and the corresponding calculated optical band gap *E*_g_ was 2.75 eV (Figure 4B, red trace). The obtained *E*_g_ values of Li_2_SnO_3_ and g-C_3_N_4_ were in excellent accordance with previous reports (Wang et al., 2012; Guo et al., 2017). Compared with the pure g-C_3_N_4_, when Li_2_SnO_3_ was composited with g-C_3_N_4_, the LSO-CN-85 heterojunction displayed a blue shift of the absorption band, which would be favorable for efficient separation of the photo-induced carriers, thus leading to a higher photocatalytic performance.

**Figure 5:**
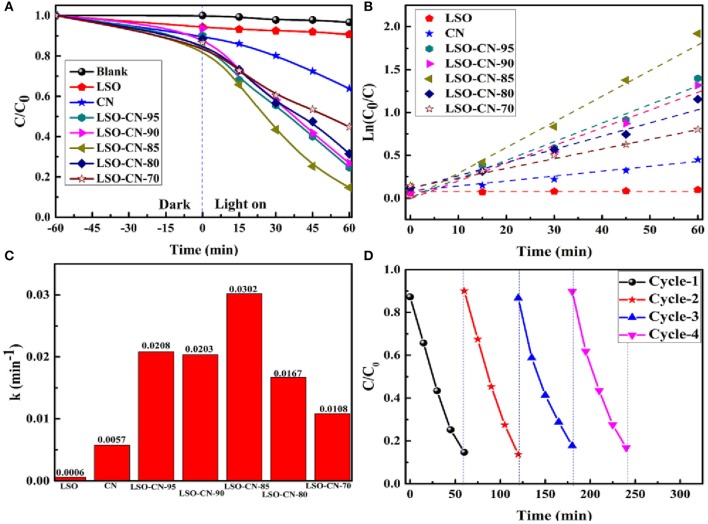
**(A)** Photocatalytic degradation of RhB with as-synthesized samples under visible light (λ ≥ 420 nm); **(B)** The pseudo-first order kinetic fitting of the photodegradation of RhB; **(C)** The fitted kinetic constants for RhB photodegradation; **(D)** Cycling experiments of LSO-CN-85 for RhB photodegradation.

The photocatalytic activities of the as-synthesized samples were evaluated by RhB photodegradation under visible light (λ ≥ 420 nm). The measured photocatalytic activities of the LSO-CN composites are presented in Figure 5. As can be seen in Figure 5A, without catalysts, the photodegradation of RhB solution under visible light was almost undetectable. The photodegradation rate in the presence of Li_2_SnO_3_ alone was only slightly higher, attributed to its wide intrinsic optical band gap. Meanwhile, pure g-C_3_N_4_ achieved the modest photodegradation rate of just 36% within 60 min irradiation. However, the photocatalytic activity of g-C_3_N_4_/Li_2_SnO_3_ was remarkably influenced by the Li_2_SnO_3_ content, and all of the LSO-CN composites exhibited superior photocatalytic activities for RhB photodegradation compared with the parent compounds g-C_3_N_4_ and Li_2_SnO_3_. Among these composites, LSO-CN-85 had the optimal photocatalytic activity, with a photocatalytic degradation efficiency of 86% under visible light within 60 min. Figure 5B presents the photocatalytic reaction kinetics of the as-synthesized samples, in which the experimental data can be described by a pseudo-first order model expressed by the following formula (Hailili et al., 2018; Xie et al., 2018):

$$\begin{array}{l} -\text{ln}\frac{C}{C_{0}}=kt \end{array}$$

where *C*_0_ and *C* are the RhB concentration in solution at time 0 and *t*, respectively. The quantity *k* is the fitted kinetic rate constant. It can be seen that the plots of the irradiation time *t* against $\text{ln}\frac{C_{0}}{C}$ are nearly straight lines, which reveals that all the photocatalysts followed pseudo-first order kinetics in the photodegradation of the RhB solution. The kinetic rate constants of Li_2_SnO_3_ and g-C_3_N_4_ were 0.0006 and 0.0057 min^−1^, respectively. For the Li_2_SnO_3_/g-C_3_N_4_ heterojunctions, the corresponding kinetic rate constants of LSO-CN-70, LSO-CN-80, LSO-CN-85, LSO-CN-90, and LSO-CN-95 were fitted as 0.0208, 0.0203, 0.0302, 0.0167, and 0.0108 min^−1^, respectively. The kinetic rate constant of the LSO-CN-85 was the highest, and was ~50 and 5 times that of pure Li_2_SnO_3_ and g-C_3_N_4_. To evaluate the stability of the photocatalytic performance, cycling experiments of the heterojunction LSO-CN-85 were carried out. As indicated in Figure 5D, the photocatalytic activity exhibited no obvious loss after four successive cycles for the photodegradation of RhB solution, and the observed XRD patterns during the cycling photocatalytic experiments still matched well with pristine LSO-CN-85 (Figure S2), both suggesting that the LSO-CN heterojunction photocatalyst was stable during the photocatalytic reaction process.

**Figure 6:**
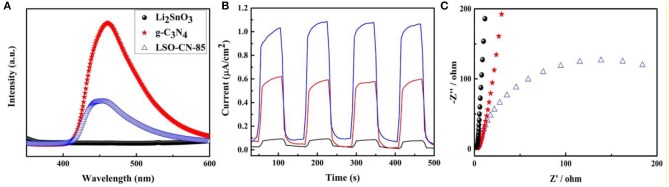
**(A)** PL spectra at the excitation wavelength of 320 nm. **(B)** Transient photocurrent responses. **(C)** EIS Nyquist plots of the as-prepared samples Li_2_SnO_3_, g-C_3_N_4_ and LSO-CN-85.

To quantify the separation efficiency of the photo-induced carriers, measurements of solid photoluminescence, photocurrent responses and electrochemical impedance spectroscopy were performed. Figure 6A presents the PL spectra of Li_2_SnO_3_, g-C_3_N_4_ and LSO-CN-85 excited at 325 nm. For Li_2_SnO_3_, no obvious emission peak was observed in the range of 400–600 nm, whereas for g-C_3_N_4_, strong fluorescence intensity was centered at ~460 nm. Generally, weaker emission intensity of a PL spectrum manifests higher separation efficiency of photo-induced carriers, implying a low recombination rate. For the heterojunction LSO-CN-85, the PL intensity was considerably lower than that of g-C_3_N_4_, indicating the strong suppression of the recombination of photo-induced carriers in the heterojunction.

Further, the photocurrent responses of the as-prepared samples were determined during four on/off visible light irradiation cycles in Na_2_SO_4_ electrolyte. As presented in Figure 6B, g-C_3_N_4_ had a markedly low transient photocurrent response because of the high recombination rate of photo-induced carriers, while Li_2_SnO_3_ exhibited the lowest photocurrent density, ascribed to its wide band gap. However, for the LSO-CN-85 heterostructure, the photocurrent density increased notably, indicating remarkably enhanced efficiency in the separation and transportation of photo-induced carriers. Next, EIS was performed to explore the conductive properties of the as-prepared samples under visible light (Figure 6C). As is well-known, in Nyquist plots, a smaller arc radius represents lower impedance and higher efficiency of charge transfer. Notably, the LSO-CN-85 heterostructure had a smaller arc radius than the parent compounds Li_2_SnO_3_ and g-C_3_N_4_, which further testified to the effective separation of photo-induced carriers after forming the heterojunction. Hence, based on the above results, the Li_2_SnO_3_/g-C_3_N_4_ heterostructure was able to promote the transfer and separation of the photo-induced carriers, leading to the enhancement of photocatalytic activity under visible light.

**Figure 7:**
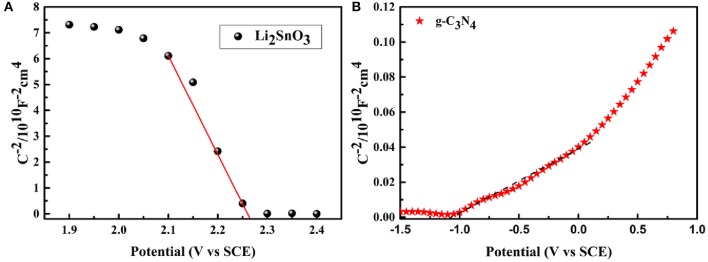
Mott-Schottky plots for **(A)** Li_2_SnO_3_ and **(B)** g-C_3_N_4_.

Mott-Schottky measurement was performed to evaluate the oxidation capability of the as-synthesized catalysts. The flat-band potentials were calculated by the Mott-Schottky equation (Gelderman et al., 2007; Cho et al., 2009; Boltersdorf et al., 2016):

$$\begin{array}{l} \frac{1}{C^{2}}=\left(\frac{2}{\varepsilon_{r}\varepsilon_{0}N_{d}e}\right)\times \left(V-V_{fb}-\frac{\kappa_{B}T}{e}\right) \end{array}$$

where *C* is the space charge capacitance, ε_*r*_ and ε_0_ are the dielectric constant of the semiconductor and the permittivity in a vacuum, *e* is the electronic charge, *N*_*d*_ is the carrier density, and *V*, *V*_*fb*_, κ_*B*_ and *T* are the applied voltage, flat-band potential, Boltzmann constant and temperature, respectively. Here, *V*_*fb*_ was obtained as the *x*-intercept of the Mott-Schottky plots ($\frac{1}{C^{2}}=0$) as a function of the applied potential. Meanwhile, the flat-band potential *V*_*fb*_ corresponds to the conduction band potential for an n-type semiconductor and the valence band edge potential for a *p-*type semiconductor. As indicated from the positive and negative slopes of the Mott-Schottky plots in Figures 7A,B, Li_2_SnO_3_ was a *p-*type semiconductor, while g-C_3_N_4_ was of the n-type. The corresponding *V*_*fb*_ of Li_2_SnO_3_ and g-C_3_N_4_ were determined to be 2.27 and −1.1 V vs. saturated calomel electrode (SCE), respectively, and these potentials relative to SCE were calibrated to the reversible hydrogen electrode (RHE) potentials through the following equation (Ke et al., 2017; Lin et al., 2018; Xu et al., 2019):

$$\begin{array}{l} V_{RHE}=V_{SCE}+0.059\;pH+V_{SCE}^{0} \end{array}$$

where *V*_*RHE*_ is the calibrated potential vs. RHE, $V_{SCE}^{0}$ equals 0.245 V, and *V*_*SCE*_ are the obtained experimental values. Thus, the *V*_*fb*_ of Li_2_SnO_3_ and g-C_3_N_4_ were 2.92 and −0.45 V vs. RHE after calibration. Herein, the flat-band potential (defined as the quasi-Fermi level) is adopted to be 0.1 V below the conduction band minimum (CBM) or above the valence band maximum (VBM) for n-type and *p-*type semiconductors, respectively. Therefore, the final VBM of Li_2_SnO_3_ and CBM of g-C_3_N_4_ were 3.02 and −0.55 V, respectively. Referring to the estimated optical band gaps from the UV-vis DRS curves, the CBM of Li_2_SnO_3_ and VBM of g-C_3_N_4_ were calculated to be −0.62 and 2.20 V, respectively.

**Figure 8:**
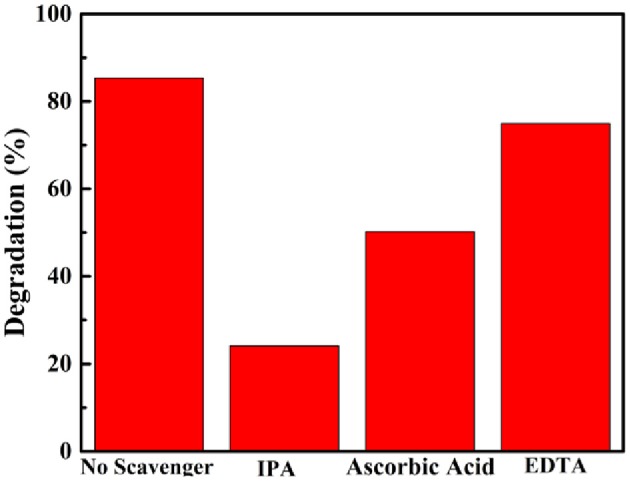
Effects of a series of scavengers on the degradation of RhB solution by heterojunction LSO-CN-85.

**Figure 9:**
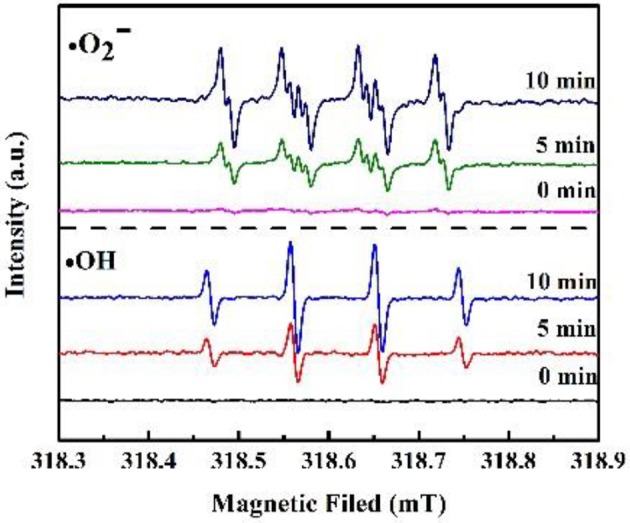
DMPO spin-trapping EPR spectra for DMPO-·OH and DMPO-·${\text{O}}_{2}^{-}$ under visible light irradiation with heterojunction LSO-CN-85.

Trapping experiments of reactive species during the photocatalytic process were carried out to explore the mechanism of the LSO-CN-85 heterojunction. As shown in Figure 8, a dramatic suppression of photodegradation efficiency was observed after adding IPA and ascorbic acid, manifesting that and were the main participants in the photocatalytic reaction. In contrast, the introduction of EDTA had only a weak influence on the photodegradation rates, demonstrating that *h*^+^ played a minor role in degrading the RhB solution. The reactive species were also detected using fluorescence spectroscopy. The increase of fluorescence intensity with prolonged irradiation time was consistent with the results of the trapping experiments (Figure S3). To further investigate the active species ·OH and ·${\text{O}}_{2}^{-}$ during the photocatalytic process, EPR measurements were performed. As presented in Figure 9, it could be seen that no EPR signal was detected in the darkness. However, the signal of ·OH and ·${\text{O}}_{2}^{-}$ were increased remarkably, when the light was on. These results further confirmed the existence of ·OH and ·${\text{O}}_{2}^{-}$ during the photocatalytic process.

**Figure 10:**
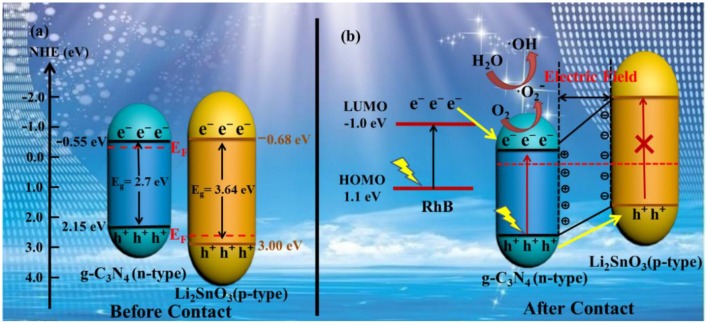
Schematic of charge transfer between *p*-type Li_2_SnO_3_ and *n*-type g-C_3_N_4_ **(a)** before contact and **(b)** after contact forming the *p-n* heterojunction.

Based on the above analysis, the proposed photocatalytic mechanism of the LSO-CN-85 heterojunction is presented in Figure 10. As revealed by the results of the Mott-Schottky measurements and UV-vis DRS experiments, the band alignments of *p-*type Li_2_SnO_3_ and g-C_3_N_4_ before formation of an interface were as presented in Figure 10a. First, when the *p-*type Li_2_SnO_3_ and g-C_3_N_4_ were combined to form the *p-n* heterostructure, the Fermi levels of Li_2_SnO_3_ tended to rise up and the Fermi levels of g-C_3_N_4_ tended to descend to reach an equilibrium state. As a result, the CB edge of Li_2_SnO_3_ was higher than that of g-C_3_N_4_ and a built-in electric field was generated in the space charge region containing negatively charged Li_2_SnO_3_ and positively charged g-C_3_N_4_ (Figure 10b). Second, once the Li_2_SnO_3_/g-C_3_N_4_ heterojunction was irradiated with visible light, photo-induced electrons and holes were generated in the g-C_3_N_4_. However, the photogenerated electrons and holes could not be excited in the Li_2_SnO_3_ owing to its intrinsic wide band gap. As a results, the inner electric field at the *p-n* heterojunction interface will push the holes in the VB of g-C_3_N_4_ toward the VB of Li_2_SnO_3_. Meanwhile, the generated electrons remained in the conduction band of g-C_3_N_4_, where the accumulated electrons reacted with O_2_ adsorbed on the surface of the heterojunction to form and, which in turn degraded RhB in the aqueous solution. Therefore, in such a way, the photogenerated electrons and holes were efficiently separated and the recombination rate was decreased. In addition, the dye sensitization effect was also considered in this system. The photoexcited electrons on the LOMO level of RhB molecule (Dong et al., 2014) were prone to transfer to the CB of g-C_3_N_4_, resulting in the increased aggregation of electrons and further enhanced the performance of the photodegradation.

## Conclusion

A novel LSO-CN heterojunction photocatalyst, comprising *p-*type Li_2_SnO_3_ and n-type g-C_3_N_4_, was successfully prepared by a facile calcining method. The obtained heterojunctions LSO-CN were characterized by PXRD, SEM, TEM, FT-IR, and UV-vis DRS. The optimum photodegradation rate was that of the heterojunction LSO-CN-85, i.e., 86% degradation of RhB after 60 min of visible light irradiation, which was ~5 times that of g-C_3_N_4_. The photo-induced and active radicals played the dominant role in the photocatalytic RhB degradation over the LSO-CN-85 heterojunction photocatalyst. Photoelectrochemical performance measurements were carried out to elucidate the photocatalytic mechanism. The enhanced photocatalytic performance could be attributed to the successful preparation of a *p-n* heterojunction between Li_2_SnO_3_ and g-C_3_N_4_, which greatly promoted the efficient separation of photo-induced carriers.

## Data Availability Statement

All datasets generated for this study are included in the article/Supplementary Material.

## Author Contributions

Specifically, YL and DY proposed this topic and design of the project. BZ and MW completed the characterization part. MW, QY, and XL completed the experimental part. YW analyzed the results. DY, BZ, and YL composed the manuscript. All authors participated in the discussions of the results and made important contributions on this work.

### Conflict of Interest

The authors declare that the research was conducted in the absence of any commercial or financial relationships that could be construed as a potential conflict of interest.
