# Supplementary material for: Novel P-n Li2SnO3/g-C3N4 Heterojunction With Enhanced Visible Light Photocatalytic Efficiency Toward Rhodamine B Degradation
Source: Front Chem. 2020 Feb 11;8:75. doi: 10.3389/fchem.2020.00075 (PMC7026459; doi:10.3389/fchem.2020.00075)
Supplement: Supplementary file 1 [file Data_Sheet_1.PDF]

Supplementary Material

Novel p-n Li_2SnO_3/g-C_3N_4 Heterojunction with Enhanced Visible Light Photocatalytic Efficiency toward Rhodamine B Degradation

Yuanyuan Li[1]*, Meijun Wu[1], Yaoqiong Wang[2], Qimei Yang[1], Xiaoyan Li[4], Bin Zhang[3]* and Dingfeng Yang[2]*

[1]Department of Biological and Chemical Engineering, Cooperative Innovation Center of Lipid Resources and Children's Daily Chemicals, Chongqing University of Education, Chongqing 400067, People`s Republic of China.

[2]College of Chemistry and Chemical Engineering, Chongqing University of Technology, 69 Hongguang Rd.,Lijiatuo, Banan District, Chongqing 400054, People`s Republic of China.

[3]Analytical and Testing Center of Chongqing University, Chongqing 401331, People's Republic of China.

[4]National ad Local Joint Laboratory of Traffic Civil Engineering Materials, Department of Materials and Engineering , Chongqing Jiaotong University, Chongqing 400074, People`s Republic of China.

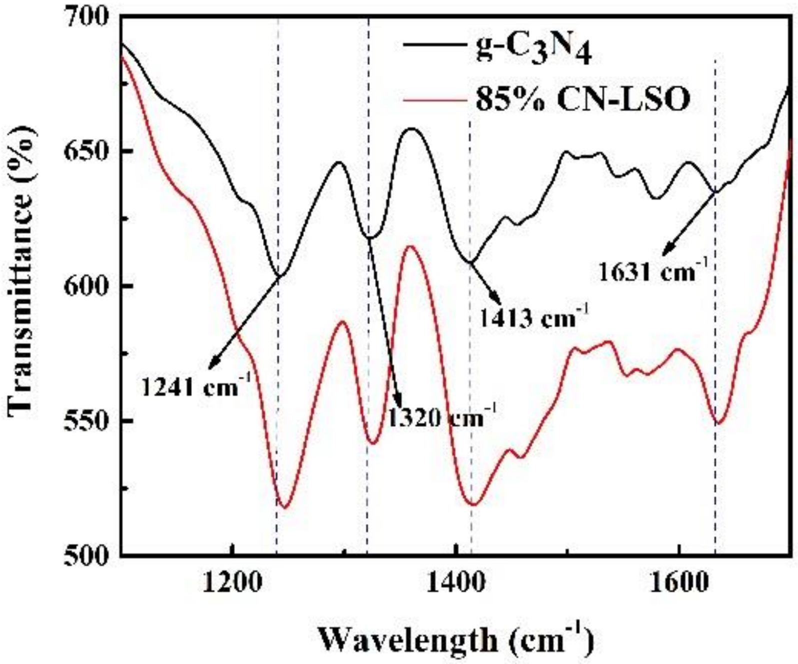

Figure S1 the magnified FT-IR spectrum of g-C_3N_4 and LSO-CN-85 heterojunction in the range of 1100 cm^{-1} to 1700 cm^{-1}.

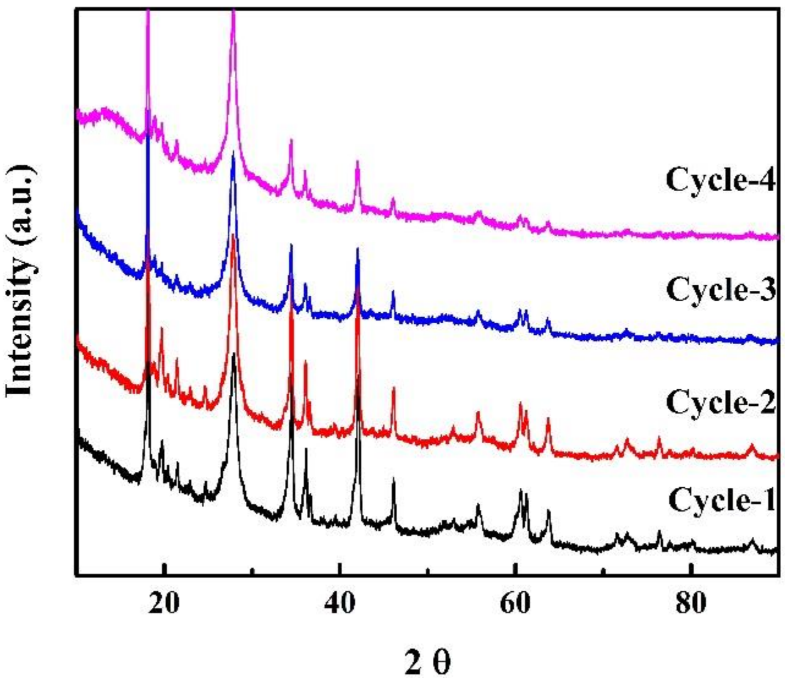

Figure S2 XRD patterns of sample LSO-CN-85 of cycling experiment on the photocatalytic reaction of degrading RhB.

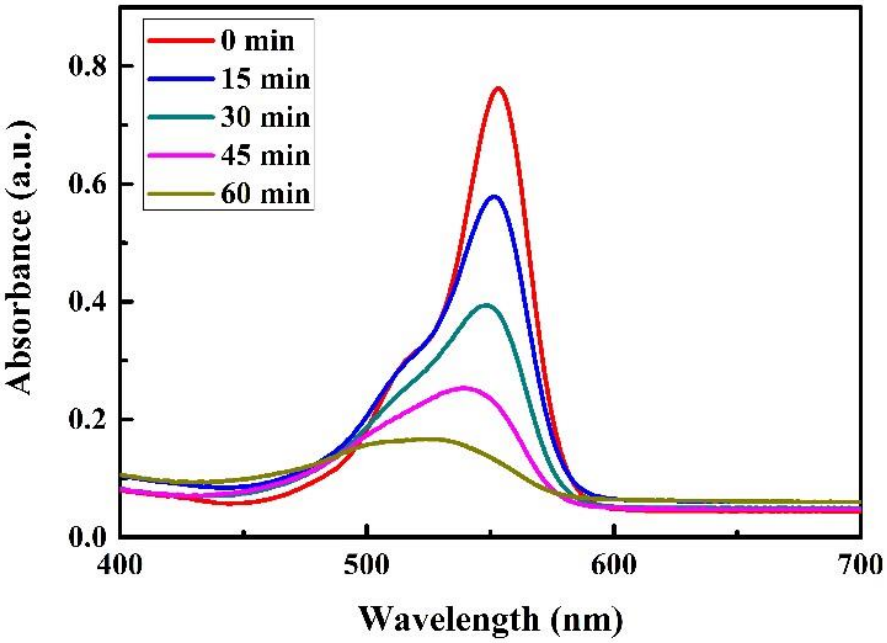

Figure S3 Hydroxyl radical ·OH detecting photoluminescence (PL) spectra of LSO-CN-85 in TA solution under UV light irradiation
